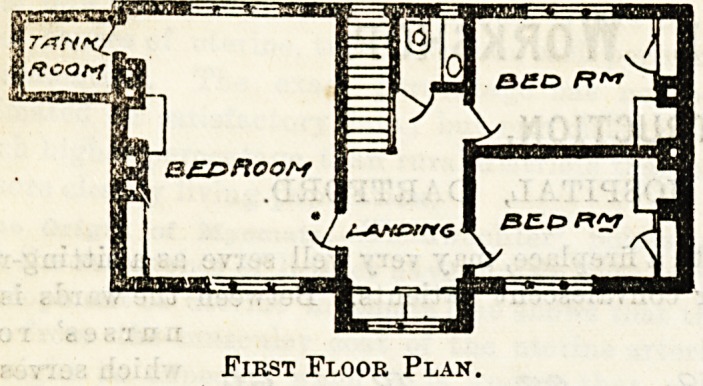# The Livingstone Cottage Hospital, Dartford

**Published:** 1895-02-16

**Authors:** 


### THE INSTITUTIONAL WORKSHOP.

### HOSPITAL CONSTRUCTION.

### THE LIVINGSTONE COTTAGE HOSPITAL, DARTFORD.

This hospital is planned on a somewhat unusual model. It consists of two distinct buildings, one being the administrative block, the other containing the wards and their offices, the two parts being connected by a covered way. There is much to be said in favour of this arrangement; it keeps the wards absolutely separate from the administration offices, and permits of a freer circulation of air than would otherwise be the case.

The administration block is partly two storeys in height, the upper storey being devoted to bed-rooms for the staff. On the ground floor the separate side entrance to the waiting-room seems to suggest out-patient work; the dispensary, if, as would appear by there being communication between it and the waiting-room, it is used as consulting-room for out-patients, is a convenient room for the purpose; otherwise it appears much too large for its work. The surgery is, we presume, the operation-room, and is provided with a top light in addition to the windows.

The ward block contains two wards of eight beds, each entered from a wide lobby which, being provided with a fireplace, may very well serve as a sitting-room for convalescent patients. Between the wards is the nurses' room, which serves the double purpose of sitting-room and duty-room. On each side of the nurses' room is a special ward for one bed. These last each communicate on one side with the nurses' room, and on the other with a lobby into which the large ward also opens, and from which access to the outside is gained. The planning of this block is not altogether satisfactory. The lighting and ventilating of the large wards is impeded by the position of the special wards and lobbies; and the nurses' duty-room, with its four doors, cannot be a very comfortable apartment. The arrangement of the ward windows is specially unfortunate, as the side where there is least window space happens in each case to be that facing south. In a small hospital like this, with accommodation for only one nurse on duty at a time, the special wards might well have been dispensed with. Indeed, it would be a practical impossibility for one nurse to manage efficiently more than the sixteen beds in the two large wards.

GROUND FLOOR PLAN.
LIVINGSTONE COTTAGE HOSPITAL, EAST HILL, DARTFORD.

The hospital was designed by Mr. G. H. Tait,

FIRST FLOOR PLAN.

a local architect, and the first stone was laid on April 21st, 1894, by Mr. H. M. Stanley, the explorer.

## Figures and Tables

**Figure f1:**
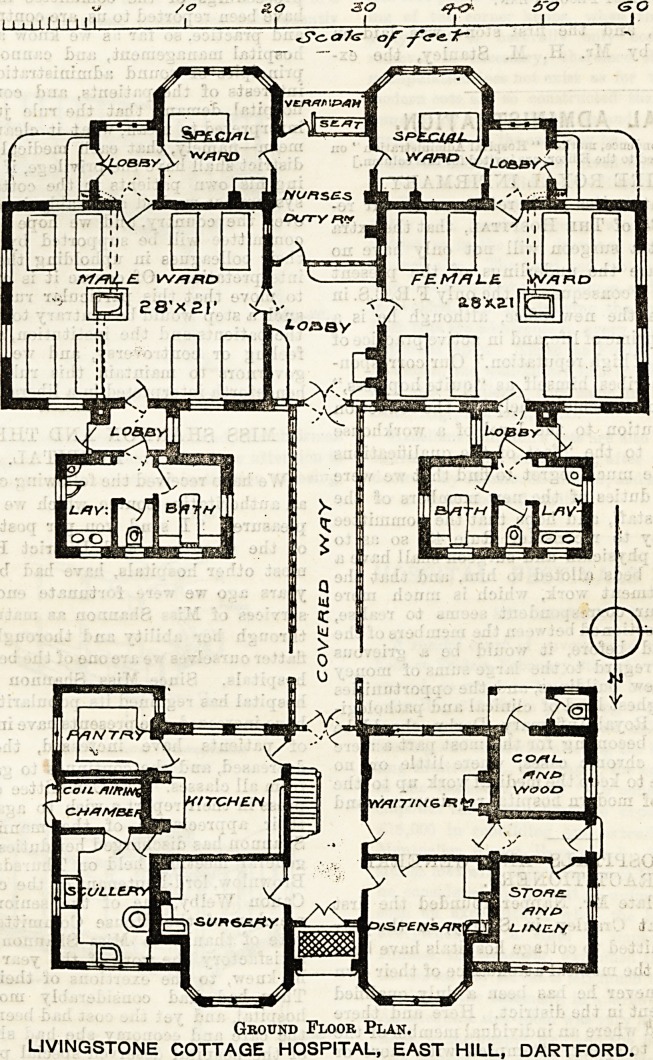


**Figure f2:**